# Age effect on the prediction of risk of prolonged length hospital stay in older patients visiting the emergency department: results from a large prospective geriatric cohort study

**DOI:** 10.1186/s12877-018-0820-5

**Published:** 2018-05-30

**Authors:** C. P. Launay, A. Kabeshova, A. Lanoé, J. Chabot, E. J. Levinoff, O. Beauchet

**Affiliations:** 1 Service of Geriatric Medicine and Geriatric Rehabilitation, Department of Medicine, Lausanne University Hospital, Lausanne, Switzerland; 2 Department of Neuroscience, Division of Geriatric Medicine Angers University Hospital, Angers, France; 3 Department of Medicine, Division of Geriatric Medicine, Sir Mortimer B. Davis - Jewish General Hospital and Lady Davis Institute for medical research, McGill University, Montreal, QC Canada; 4 Dr. Joseph Kaufmann Chair in Geriatric Medicine, Faculty of Medicine, McGill University, Montreal, QC Canada; 5 Centre of Excellence on Aging and Chronic Diseases of McGill University Health Network, Montreal, QC Canada

**Keywords:** Length of hospital stay, Prediction, Elderly

## Abstract

**Background:**

With the rapid growth of elderly patients visiting the Emergency Department (ED), it is expected that there will be even more hospitalisations following ED visits in the future. The aim of this study was to examine the age effect on the performance criteria of the 10-item brief geriatric assessment (BGA) for the prolonged length of hospital stay (LHS) using artificial neural networks (ANNs) analysis.

**Methods:**

Based on an observational prospective cohort study, 1117 older patients (i.e., aged ≥ 65 years) ED users were admitted to acute care wards in a University Hospital (France) were recruited. The 10-items of BGA were recorded during the ED visit and prior to discharge to acute care wards. The top third of LHS (i.e., ≥ 13 days) defined the prolonged LHS. Analysis was successively performed on participants categorized in 4 age groups: aged ≥ 70, ≥ 75, ≥ 80 and ≥ 85 years. Performance criteria of 10-item BGA for the prolonged LHS were sensitivity, specificity, positive predictive value [PPV], negative predictive value [NPV], likelihood ratios [LR], area under receiver operating characteristic curve [AUROC]). The ANNs analysis method was conducted using the modified multilayer perceptron (MLP).

**Results:**

Values of criteria performance were high (sensitivity> 89%, specificity≥ 96%, PPV > 87%, NPV > 96%, LR+ > 22; LR- ≤ 0.1 and AUROC> 93), regardless of the age group.

**Conclusions:**

Age effect on the performance criteria of the 10-item BGA for the prediction of prolonged LHS using MLP was minimal with a good balance between criteria, suggesting that this tool may be used as a screening as well as a predictive tool for prolonged LHS.

## Background

A growing number of older adults (i.e., age 65 and over) visit the emergency departments (EDs) [1]. In Europe, they account for around 20% of all EDs visitors [1, 2]. These older ED visitors, particularly the oldest group (i.e., age 85 and over), generally have a longer length of hospital stay (LHS) after their ED discharge to acute care wards compared to younger ED visitors [1–3]. The high morbidity burden and related-disabilities expose older patients to an increased risk of non-fatal health outcomes like a long LHS [2, 4, 5]. With the rapid growth of the oldest segment of ED visitors, hospitalization after an ED admission is expected to be even greater in the future and, thus, hospitals need to confront this new challenging issue [1–3, 6].

One way to reduce LHS is early identification of older ED visitors at greater risk of prolonged LHS after an ED discharge to acute care wards [1, 5, 6]. This screening is a crucial step for targeting appropriate interventions to prevent or decrease the occurrence of non-fatal health outcomes. The predictive tools designed for this purpose should provide a relevant stratification of risk and give information early; ideally before the hospital admission in order to avoid or plan the admission [4, 7]. The use of clinical information collected by a physician has been shown to be the best strategy to develop predictive tools of unplanned hospital admissions compared to self-reported and administrative data collection [8, 9] A limited number of studies have used tools aimed at identifying older patients at greater risk of prolonged LHS after an ED visit, with low predictive accuracy [2, 3, 5, 6]. Recently, the 10-item Brief Geriatric Assessment (BGA), was reported to have a high specificity (97%) but a lower sensitivity 63% [10]. This study reported the best criteria performance to date. This result was explained in part by the use of artificial neural networks (ANNs), and in particular the modified multilayer perceptron (MLP) [10]. Indeed, ANNs analysis is particularly adapted to predict an inherent complex event like prolonged LHS [11, 12]. The main limit of this previous study was the unbalance between sensitivity and specificity, which could be related to the high amount of data required by ANNs [11, 12]. In addition, because the risk of hospitalization increases with age, it could be suggested that the best balance with greater values of performance criteria could be reported specifically in the oldest age group (i.e., age 85 and over) of ED users [1–4]. The reported study aims to examine the effect of age on the predictive abilities (i.e., sensitivity, specificity, positive predictive value [PPV], negative predictive value [NPV], likelihood ratios [LR], area under receiver operating characteristic curve [AUROC]) of the 10-item BGA for the prolonged LHS using MLP in geriatric ED visitors.

## Methods

### Participants

A total of 1117 older patients (i.e., aged ≥ 65 years) were recruited upon their hospitalization after an ED visit in a University Hospital (France) from January 2013 and December 2013. This study is an ongoing study which began in 2011 and its procedure for participant’s recruitment has been previously described in detail [6, 10]. To be included, patients had to be hospitalized on acute care wards after an ED visit, age 65 years and over, and willingness to participate in research. Patients who died during hospitalization were excluded.

### Assessment

The 10-item BGA was fulfilled upon admission to the ED and was composed of the following items: age ≥ 85 years, male gender, polypharmacy defined as ≥5 drugs per day, use psychoactive drugs (i.e., benzodiazepines, antidepressants or neuroleptics), history of falls in previous 6 months, temporal disorientation (i.e., inability to give the month and/or year), presence of acute organ failure plus reason for admission, living situation (home versus institution), and non-use of formal and/or informal home-help services. The nature of the acute organ failure for ED visit was categorized in five groups: cardio-vascular diseases, respiratory diseases, digestive diseases, neuropsychiatric diseases, and other acute diseases (Table 1). Other acute diseases referred to a heterogenous groups of diseases including traumatic injuries, hepatic failure, hematological failure and kidney failure.

**Table 1:** The 10-item Brief Geriatric Assessment

Items	Yes	No
Age ≥ 85 years		
Male gender		
≥ 5 drugs per day		
Use psychoactive^a^ drugs		
History of falls in the past 6 months		
Temporal disorientation^b^		
Acute organ failure		
Reason for admission:		
Cardio-vascular diseases		
Respiratory diseases		
Digestive diseases		
Neuropsychiatric diseases		
Other acute diseases		
Living situation		
Home		
Institution		
Non-use of formal and/or informal home-help services		

^a^hypnotics, anti-depressants or neuroleptics

^b^unable to give the current year and/or month

### Outcome measure

The LHS was calculated using the administrative registry of the University Hospital and corresponded to number of days between the first day of ED visit and the last day of hospitalization on an acute care ward. Prolonged LHS was defined as being in the top third of LHS, which corresponded to more than 13 days in the studied sample. The main issue to identify this threshold value is that there is no consensus on the definition of a prolonged length of hospital stay is in geriatric acute care unit. The absence of definition is due to the fact that a prolonged length of hospital stay depends on an accumulation and complex interplay between several variables. These variables are related to the health status of patients but also to the environment where they are hospitalized (e.g., flux of patients, number of health professionals, type of hospital, organization of care etc.…). Thus, the unique solution to determine this threshold is to use the consensus methods of tertilization [6, 10].

### Standard protocol approvals, registrations, and participant consents

Patients recruited in this study provided themselves a verbal consent or received help from their trusted person. The consent to participate was recorded in the patients’ digital files. Ethical Committee of Angers, France, approved the entire procedure.

### Statistical analysis

**Fig. 1:**
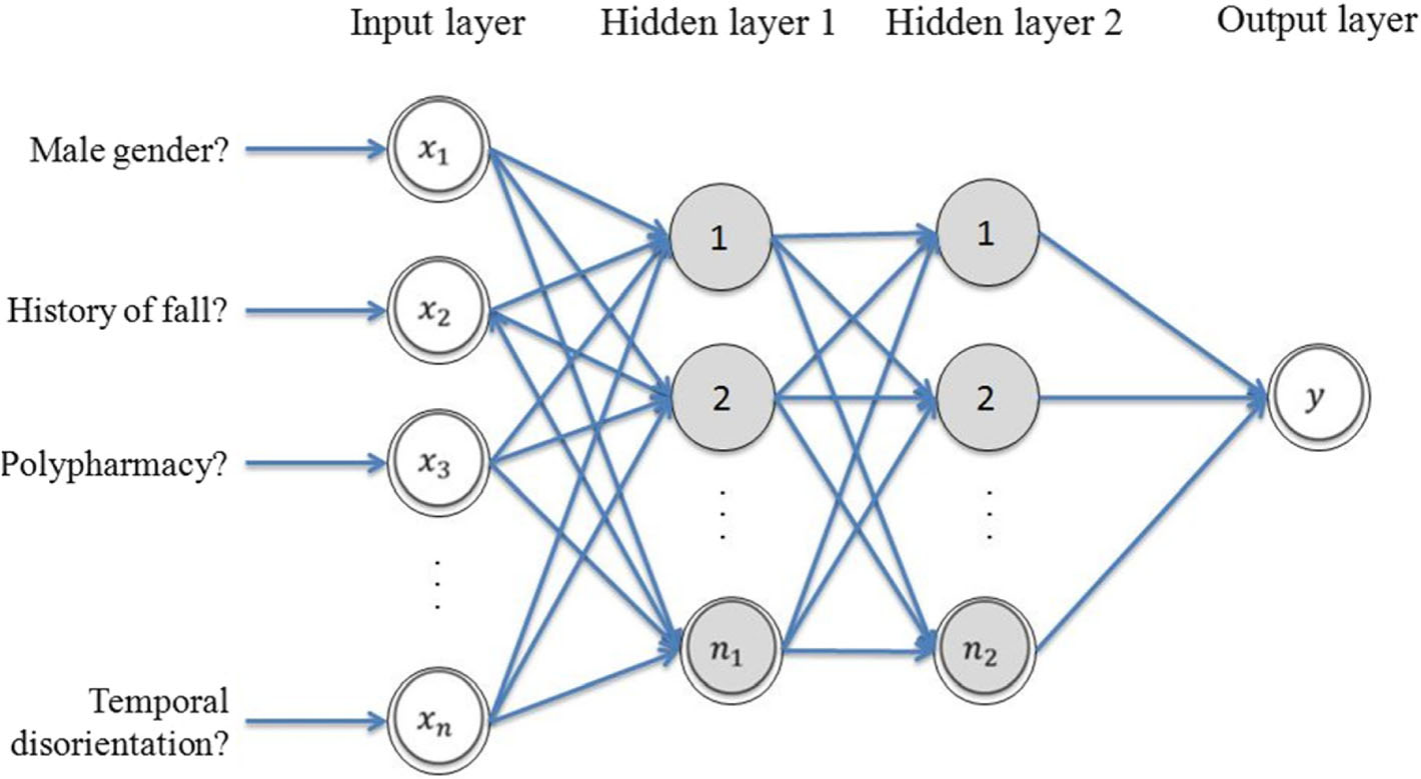
General structure of modified multilayer perceptron in this study

Participants were split into two subgroups based on the presence or absence of a prolonged LHS. The top third of LHS defined the prolonged LHS (i.e., > 13 days). Univariate logistic regression models were used to examine the association between prolonged LHS (dependent variable) and 10-item BGA (independent variables). Artificial neural networks (ANNs) are inspired by animals’ brain and provide computational processing based on machine learning. ANNs are more appropriate to examine “chaotic” events, such as prolonged LHS, because they are not linear statistical models. These systems are interconnected and composed of multiple layers. Nodes from one layer are connected to all nodes in the following layer, but there were no lateral connections within the layer (Fig. 1). The output layer comprised one neuron, indicating the presence or absence of prolonged LHS.The “*neuralnet: Training of neural networks*” R package was used for Modified multilayer perceptron (MLP) combining with a specific algorithm (9, 10). To perform ANNs analysis, the sample of participants was randomized in two subgroups (i.e., a training group and a testing group). There was no significant difference between training and testing group (data not shown). Between-group comparisons were performed using unpaired *t*-test, Pearson’s Chi-squared test with Yates’ continuity correction, as appropriate. Four age groups were identified: ≥ 70, ≥ 75, ≥ 80 and ≥ 85 years old. Performance criteria were sensitivity, specificity, PPV, NPV, LR+, LR- and AUROC. All statistics were performed using R 3.1.0 and Net Beans IDE 8.0.

## Results

There was a trend for a greater mean age (*P* = 0.0699), a greater prevalence of temporal disorientation (OR = 2.65, *P* < 0.001) in participants with prolonged LHS compared to those with short LHS (Table 2). In addition, participants with prolonged LHS visited the ED less often for digestive diseases (OR = 0.48, *P* = 0.0189) and more often for other diseases (OR = 1.46, *P* = 0.089) compared to those with short LHS. The mean LHS was 21.6 ± 8.8 days for older ED users with prolonged LHS and 5.2 ± 3.7 days for those who had no prolonged LHS. Whatever the age group considered, predictive performance were high(sensitivity> 89%, specificity≥ 96%, PPV > 87%, NPV > 96%, LR+ > 22; LR- ≤ 0.1 and AUROC> 93). Participants over 75 years showed the best performance (sensitivity = 89.7%, specificity = 97.8%, PPV = 93.4, NPV = 96.5, LR + =41.0; LR- = 0.1 and AUROC = 93.7) (Table 3).

**Table 2:** Baseline characteristics of participants separated in training and testing groups and univariate logistic regression showing the association between prolonged length of hospital stay (dependant variable) and 10-item Brief Geriatric Assessment (independent variables). (*n* = 1117)

Characteristics	Prolonged length hospital stay (i.e., > 13 days)				*P*-Value*
	No (*n* = 840)	Yes (*n* = 277)	OR	[95% CI]	
Age (years)					
Mean ± SD	85.14 ± 5.97	85.13 ± 5.62			0.0699
≥ 70 years	840 (100.0)	276 (99.6)	–	–	0.5593
≥ 75 years	834 (99.3)	271 (97.8)	0.325	[0.100;1.074]	0.0898
≥ 80 years	672 (80.0)	230 (83.0)	0.268	[0.267;0.292]	0.3066
≥ 85 years	463 (55.1)	150 (54.2)	0.779	[0.779;0.781]	0.8329
Male gender, *n* (%)	351 (41.8)	114 (41.2)	0.975	[0.738;1.283]	0.9090
Number of drugs daily taken					
Mean ± SD	6.52 ± 3.22	6.33 ± 3.33	–	–	0.6094
≥ 5, *n* (%)	601 (71.5)	206 (74.4)	1.152	[0.849;1.577]	0.4055
Use of psychoactive drugs^a^, *n* (%)	398 (47.4)	139 (50.2)	1.267	[0.965;1.664]	0.1516
History of falls during the past 6 months, *n* (%)	516 (61.4)	175 (63.2)	1.122	[0.848;1.491]	0.6540
Temporal disorientation^b^, *n* (%)	259 (30.8)	150 (54.2)	**2.652**	**[2.009;3.510]**	**< 0.0001**
Non-use of formal and/or informal home-help services^c^	576 (68.6)	196 (70.8)	1.084	[0.807;1.464]	0.6828
Acute organ failure	503 (59.9)	178 (64.3)	1.204	[0.909;1.600]	0.2207
Living at home	594 (70.7)	198 (71.5)	1.037	[0.770;1.406]	0.8673
Acute organ failure as reason for admission to Emergency Department, n (%)					
Cardio-vascular diseases, *n* (%)	92 (11.0)	37 (13.4)	1.256	[0.825;1.880]	0.3282
Respiratory diseases, *n* (%)	98 (11.7)	23 (8.3)	0.689	[0.418;1.091]	0.1469
Digestive diseases, *n* (%)	79 (9.4)	13 (4.7)	**0.479**	**[0.250;0.848]**	**0.0189**
Neuropsychiatric diseases, *n* (%)	121 (14.4)	30 (10.8)	0.724	[0.466;1.095]	0.1593
Other diseases, *n* (%)	450 (53.6)	174 (62.8)	**1.463**	**[1.108;1.940]**	**0.0089**

*P*-value significant (i.e., *P* < 0.05) in bold

*OR* Odds Ratio, *CI* Confidence Interval, *SD* standard deviation

*Comparison based on unpaired t-test or Pearson’s Chi-squared, as appropriate

^a^Use of benzodiazepines or antidepressants or neuroleptics

^b^Inability to give the month and/or year

^c^Formal (i.e., health and/or social professional) or informal (i.e., family and/or friends)

**Table 3:** Performance criteria of 10-item brief geriatric assessment for the prediction of prolonged length hospital stay^a^ using artificial neural networks (i.e.; modified multilayer perceptron) based on age categories of participants (*n* = 1117)

Age categories^b^	Sensitivity	Specificity	PPV	NPV			AUROC	Number of individuals classified			
	(%)	(%)	(%)	(%)	LR+	LR-					
								TP	FP	FN	TN
≥ 70 years	91.0	96.0	87.7	97.1	22.7	0.1	93.8	242	34	24	816
≥ 75 years	89.7	97.8	93.4	96.5	41.0	0.1	93.7	253	18	29	805
≥ 80 years	91.2	96.5	89.6	97.0	25.7	0.1	93.2	206	24	20	652
≥ 85 years	90.0	96.8	90.0	96.8	27.8	0.1	95.5	135	15	15	448

*PPV* Positive predictive value, *NPV* Negative predictive value, *LR+* Likelihood ratio of positive test, *LR-* Likelihood ratio of negative test, *AUROC* Area under receiver operating characteristic curve, *TP* True positive, *FP* False positive, *TN* True negative, *FN* False negative

^a^Defined as being in the highest tertile of length of hospital stay (i.e., > 13 days)

^b^only combinations involving at least 10 participants were considered

## Discussion

The findings show that effect of age was minimal on predictive abilities of the 10-item BGA. These results suggest that analysis provided by ANNs may enable to use 10-item BGA as a screening tool but also as a predictive tool to identify older patients at higher risk of prolonged LHS, whatever their age.

The best criteria performances for prolonged LHS were shown with patients aged 75 years and over. This is an unexpected finding because it was hypothesized that greater values of criteria performance could be reported in the oldest segment of ED users. This result is discordant with previous studies which reported a strong association between age and the risk of prolonged LHS [1–4]. Age has previously been identified as an important predictor for prolonged LHS [10, 13, 14]. For instance, in a similar sized cohort of patients admitted to ED (993 patients, mean age = 87.04 years) age and gender explained 21.6% of area under receiver operating characteristic curve value [10]. In the same way, Campbell et al. reported in a larger cohort of patients admitted to ED (1626 patients, mean age = 78.7 years) that age over 85 years was strongly associated with prolonged LHS (OR = 7.6, *P* < 0.001) [14]. The association between increased age and prolonged LHS has been explained by incident disabilities that exceed 50% in hospitalized patients aged 85 years and over [14–16]. This finding is consistent with Sourial et al. who showed that age and gender had the highest contribution (C statistic values from 0.51 to 0.67) in predictive accuracy of incident disability in a cohort composed of 6657 patients (mean age = 73.68 years) [17].

A possible explanation of the discordance about age effect on the prediction of prolonged LHS shown in our study compared to previous studies could be related to the profile of population recruited, which is oldest old patients with a mean age around 85 years. Moreover, ANNs provide a different statistical approach that consider the complex interplay between all items [11, 12, 18]. Indeed, previous results of ANNs reported that using numerous variables increased predictive accuracy (area under cover values lie between 84.1 with 9 items and 90.5 with 10 items) but also modified the contribution of demographic items in the predictive performance (from 12.8% with 9 items to 21.3% with 10 items) [10]. Categorizing age groups provide an additional variable that limits the analysis of ANNs to a single age group and may modify the distribution and weight of 10 items in contribution of the predictive accuracy. Thus, ANNs take into account the variations in the contribution of all types of variables (demographic items, acute or chronic diseases, and environmental items) to increase predictive performance and to learn to recognize patterns of prolonged LHS in each age group.

Our findings underscored that regardless of age, values of criteria performance were high (sensitivity> 88%, specificity≥ 96%, PPV > 87%, NPV > 96%, LR+ > 22; LR- ≤ 0.1 and AUROC> 93). To the best of our knowledge, the current study demonstrates the best performance and balance between criteria reported for prediction of LHS after an ED visit. This result is discordant from a recent previous study which reported lower values and an unbalance between sensitivity and specificity [10]. The main explanation as suggested in our hypothesis could be related to a difference in the number of participants. In our study, we included 1117 individuals which likely increased the accuracy of prediction. It has been shown that ANNs may provide accurate information on an event only if there is a sufficient quantity of data points to be analysed [11, 12].

In order for a screening test to be applicable, it requires high level sensitivity to limit false-negative results. With sensitivity above 89% in all age group conditions, the 10-items BGA is a useful screening tool that can be applied to identify early older ED users at higher risk of prolonged LHS after their discharge to acute care wards. In addition, our results demonstrated a high specificity above 95%, which implies that there is a low false positive rate when applying the 10-items BGA to predict length of hospitalization. Thus, the combination of both high sensitivity and specificity indicates that the 10-items BGA is not only a simple screening tool but also a diagnostic tool with excellent predictive accuracy. The ability to analyse our data with the use of ANNs methods analysis is the main explanation for these findings. Indeed, ANNs are data analysis tools developed to overcome the limitations of traditional linear models as a method to predict health events [18]. ANNs are computational models which are capable of machine learning and pattern recognition [7–12]. Because they apply non-linear statistics to pattern recognition, ANNs are particularly adapted to “chaotic” events like prolonged LHS. Nowadays, the advances generated by ANNs combined with improvement of computer technology affords the opportunity to explore new perspectives using ANNs as decision-making diagnostic aids for physicians. Thus, these results can be applied directly to clinical practice because they can be developed as software applications for computers and hand held devices. The 10-item BGA may provide answers to facilitate clinical decision-making process because this tool provides a risk stratification of patients at risk of non-fatal health outcomes. Such information may be relevant to make the right decision for the patients like the discharge to home or to a medical ward, and to continue the appropriate interventions in the right patients and at the right time by the right professionals (i.e., geriatric intervention versus no geriatric intervention).

Our results also showed that patients with digestive diseases had a shorter LHS compared to patients admitted for other diseases. One explanation could be that admissions for digestive diseases are more often semi-urgent or non-urgent [19]. This low degree of urgency may explain a lower LHS. In contrast, “Other diseases” as a reason for admission was associated with an increases LHS. This group refers in part to traumatic injuries related to falls. Unlike young patients, the most common mechanism for traumatic injuries in older patients is due to fall [20]. Falls have been identified as major cause of unintentional injury leading to prolonged LHS and death, especially over 80 years, that could explain our results [21].

The strengths of this study include the large number of participants, the prospective cohort design, the hard outcome represented by prolonged LHS, and the use of sophisticated new statistical models. However, limitations need to be considered including recruitment of participants from a single center and the fact that important items related to prolonged LHS could have been forgotten. Besides, we included inpatients who died during their hospitalization and those discharged in another hospital. Both date of death or transfer to another hospital were considered as the last day of hospitalization. Thus, a bias might exist because of a suspected higher complexity of those patients.

## Conclusion

Age stratification provided minimal effect on the abilities of the 10-item BGA to predict prolonged LHS using ANNs. Indeed, ANNs provided homogeneous predictive performance that enable to use 10-item BGA as a screening tool but also as a predictive tool to identify older patients at higher risk of prolonged LHS, whatever their age.

## Abbreviations

ANNs: Artificial neural networks
AUROC: Area under receiver operating characteristic curve
ED: Emergency department
LHS: Length of hospital stay
LR: Likelihood ratio
MLP: Modified multilayer perceptron
NPV: Negative predictive value
PPV: Positive predictive value
